# WHSC1L1 drives cell cycle progression through transcriptional regulation of CDC6 and CDK2 in squamous cell carcinoma of the head and neck

**DOI:** 10.18632/oncotarget.9897

**Published:** 2016-06-07

**Authors:** Vassiliki Saloura, Theodore Vougiouklakis, Makda Zewde, Kazuma Kiyotani, Jae-Hyun Park, Guimin Gao, Theodore Karrison, Mark Lingen, Yusuke Nakamura, Ryuji Hamamoto

**Affiliations:** ^1^ Department of Medicine, University of Chicago, Chicago, IL, USA; ^2^ Department of Public Health Sciences, University of Chicago, Chicago, IL, USA; ^3^ Department of Pathology, University of Chicago, Chicago, IL, USA; ^4^ Department of Surgery, University of Chicago, Chicago, IL, USA

**Keywords:** WHSC1L1, squamous cell carcinoma of the head and neck, CDC6, CDK2, H3K36me2

## Abstract

Wolf-Hisrchhorn Syndrome Candidate 1-Like 1 (WHSC1L1) is a protein lysine methyltransferase that is recurrently amplified (8p11.23) in patients with squamous cell carcinoma of the head and neck (SCCHN). In this study, we investigated the oncogenic role of WHSC1L1 in SCCHN. Using immunohistochemistry on tissue microarrays of patients with locoregionally advanced SCCHN, we found that WHSC1L1 is significantly overexpressed in patients with SCCHN, and is associated with poor grade and heavy smoking history. Knockdown of WHSC1L1 expression resulted in significant growth suppression and reduction of H3K36 dimethylation (H3K36me2) in SCCHN cells. Chromatin immunoprecipitation analysis showed that WHSC1L1 and H3K36me2 are enriched in the gene bodies of the cell cycle-related genes *CDC6* and *CDK2*, implying that WHSC1L1 directly regulates the transcription of these genes. According to the importance of *CDC6* and *CDK2* for G1 to S transition, WHSC1L1 knockdown induced strong G0/G1 arrest which was rescued by introduction of wild-type WHSC1L1 but not by that of enzyme-inactive WHSC1L1. Our results imply that WHSC1L1 and its product H3K36me2 are essential for the transition from G1 to S phase in SCCHN cells and that WHSC1L1 could serve as a rational target for anticancer drug development for patients with head and neck cancer.

## INTRODUCTION

Squamous cell carcinoma of the head and neck (SCCHN) is a significant public health concern estimated to affect over 48,000 patients in 2016 in the United States [1]. Chemoradiotherapy, cetuximab and platinum-based chemotherapy are standard treatments [2] but many patients eventually develop resistance through escape pathways. Research efforts in the past three decades have been focusing on the investigation of tyrosine kinase inhibition and immunotherapy with checkpoint blockade, which show promise in a personalized context. Although a significant proportion of patients have temporary responses, most of them eventually progress through these treatments and die of this disease. Thus, the continued search for novel targets for anti-cancer drug development is mandatory and truly urgent.

The Cancer Genome Atlas Consortium (TCGA) [3, 4] has revealed a plethora of genetic and expression alterations in a class of enzymes known as protein methyltransferases (PMTs) in many different cancer types, suggesting the importance of these enzymes in cancer development and progression [5]. Specifically in SCCHN, genetic analysis of 530 tumors showed mutations, amplifications and deletions in approximately 68% of the samples. Interestingly, amongst the top two most frequently altered enzyme families in SCCHN were the NSD (nuclear SET-*S*uppressor of variegation 3-9, *E*nhancer of zeste and *T*rithorax-domain, NSD1, NSD2/WHSC1/MMSET, NSD3/WHSC1L1) family of protein lysine methyltransferases (PKMTs) which are genetically altered in 28% of SCCHN, implying a critical role for these enzymes in SCCHN oncogenesis [3, 4]. A similar frequency of genetic alterations in NSD PKMTs is also observed in lung squamous cell carcinoma, a disease with very similar genetic background as SCCHN. Among these enzymes, TCGA reported a statistically significant, recurrent amplification of *WHSC1L1* at chromosome 8p11.23 region in 9.3% of SCCHN tumors (Q-value=3.7e-15). Importantly, *WHSC1L1* seems to be the only recurrently amplified PKMT in SCCHN. In addition, no studies have investigated the role of WHSC1L1 in the pathogenesis of SCCHN.

WHSC1L1, also known as Wolf-Hirschhorn syndrome candidate 1-like 1, is a nuclear protein mapped at chromosome 8p11.23 and is known to function as a chromatin modifier by modulating the expression of genes through dimethylation of lysine 36 on histone H3 (H3K36me2). It shares 70-75% homology in amino-acid sequence with the other two members of the NSD family and contains a SET domain that possesses methyltransferase activity [6, 7]. There are two major WHSC1L1 isoforms, the long WHSC1L1 (1437aa) and the short WHSC1L1 (645aa), which share a common 620aa N-terminal region, while the short WHSC1L1 lacks the SET domain.

Given that WHSC1L1 is recurrently amplified in SCCHN and that few studies have explored the role of PKMTs in SCCHN [8], we aimed to evaluate its potential function as an oncogenic force in this disease, and to elucidate relevant mechanisms of its oncogenic activity. In this study, we show that WHSC1L1 is significantly overexpressed in patients with SCCHN, its knockdown causes cell-cycle arrest and decrease in global H3K36 dimethylation levels, and that it promotes the coordinated transcription of a number of cell cycle genes, including CDK2 and CDC6, which are critical for the G1/S transition. These results highlight the promising role of WHSC1L1 as a therapeutic target in SCCHN.

## RESULTS

### WHSC1L1 is overexpressed in squamous cell carcinoma of the head and neck and is associated with poor grade and smoking

**Figure 1:**
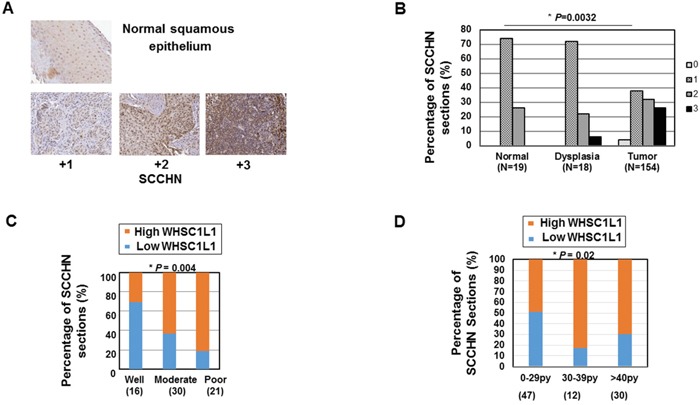
WHSC1L1 is significantly overexpressed in SCCHN and is associated with poor grade and heavy smoking history **A.** Immunohistochemistry of WHSC1L1 in normal epithelium and SCCHN tumors. WHSC1L1 is mostly expressed in the nucleus of normal squamous and SCCHN samples. Mild expression of WHSC1L1 was also observed in the cytoplasm. **B.** WHSC1L1 is significantly overexpressed in SCCHN sections compared to normal and dysplastic epithelium and the percentage of sections with mild staining (+1) decreased, while the percentage of sections with strong staining (+3) increased significantly from normal epithelium to dysplasia and from dysplasia to squamous cell carcinoma (*P*=0.0032, Cochrane-Armitage test). **C.** High WHSC1L1 expression correlates with poor grade (*P*=0.004, logistic regression). **D.** High WHSC1L1 expression correlates with heavier smoking history (*P*= 0.021, logistic regression).

To assess the expression pattern of WHSC1L1 in patients with SCCHN, immunohistochemistry (IHC) using tissue microarrays consisting of tumor from 154 patients with local or locoregionally advanced SCCHN and 19 samples of adjacent normal squamous epithelial tissue as baseline reference was performed. Clinical information was also available for 105 patients examined for WHSC1L1. Figure 1A shows representative examples of SCCHN sections stained at different IHC scores (4-scale grading: 0, +1, +2, +3), with normal buccal mucosal epithelium demonstrating only weak staining in the nucleus of the cells. WHSC1L1 was localized predominantly in the nucleus, but weak staining was also observed in the cytoplasm. WHSC1L1 was significantly overexpressed in SCCHN (n=132) compared to normal squamous (n=19) and dysplastic (n=18) epithelium (*P*=0.0074 and *P*=0.022 respectively, Wilcoxon Rank sum test). Similarly, the percentage of samples with strong (+3) expression of WHSC1L1 increased significantly from 0% to 6% from normal to dyplastic epithelium, and then to 25% in SCCHN (*P*=0.0032, Cochrane-Armitage test) (Figure 1B). IHC scoring revealed moderate or strong nuclear staining (+2, +3) in 58% of SCCHN samples, and absent or weak (0, +1) staining in 42% of the samples, while 74% of the normal epithelium samples stained weakly for WHSC1L1.

Given that WHSC1 was also reported to be significantly overexpressed in SCCHN [14], we assessed the correlation between expression levels of WHSC1L1 and WHSC1 in SCCHN samples and found them to be only mildly correlated (Spearman correlation co-efficient *ρ*=0.37, *P*<0.001), suggesting that WHSC1 and WHSC1L1 may function as independent drivers of SCCHN oncogenesis.

We then proceeded to assess correlations of WHSC1L1 expression with various clinicopathological parameters, such as overall and progression-free survival, age, gender, primary site, tumor size (T), nodal stage (N), TNM stage, grade, smoking history and human papilloma virus status in SCCHN patients with local or locoregionally advanced disease who were treated with either surgery with or without adjuvant chemoradiation, or definitive chemoradiation. A summary of the data is shown in Table 1. The analysis revealed significant correlation of high WHSC1L1 expression levels (+2, +3 IHC score) with poor differentiation grade (Figure 1C) and heavier smoking history (Figure 1D). More specifically, 81% of patients with poor differentiation grade had significantly higher WHSC1L1, compared to 31% of patients with well differentiated SCCHN (logistic regression, *P*=0.004). In addition, 70% of patients with heavy smoking history (>40 pack/years) had significantly higher WHSC1L1 staining compared to 49% of patients with less than 29 pack/years of smoking history and this difference was statistically significant (logistic regression, *P*=0.021). No statistically significant associations with age, gender, primary site, HPV status, tumor stage, or nodal stage were found. No associations with overall and progression-free survival were found either, though this study was not powered to detect survival differences.

**Table 1:** Clinicopathological correlations of dichotomized WHSC1L1 expression by IHC in locoregionally advanced SCCHN

Clinicopathological Parameters	WHSC1L1		*P* value
	Low (0,+1) (n = 41)	High (+2,+3) (n = 64)	
Gender			0.45
Female	10	20	
Male	31	44	
Age (mean)	57	57.5	0.52
Primary site			0.64
Hypopharynx	6	11	
Larynx	9	17	
Tongue	10	9	
Tonsil	16	24	
Smoking status^1^			0.021
0-29py	24	23	
30-39py	2	10	
>=40py	9	21	
Grade^1^			0.004
Well differentiated	1	5	
Moderately differentiated	11	19	
Poorly differentiated	4	17	
Stage^1^			0.26
I	6	4	
II	0	1	
III	4	4	
IVA/B	31	50	
T^1^			0.69
T1	9	15	
T2	7	14	
T3	7	9	
T4	15	20	
N^1^			0.41
N0	17	13	
N1	2	8	
N2	20	37	
N3	2	4	
HPV status^1^			0.68
p16+	17	12	
p16-	36	21	

1 Patients with missing data excluded

### WHSC1L1 is overexpressed in squamous cell carcinoma cell lines and its knockdown decreases cell viability

**Figure 2:**
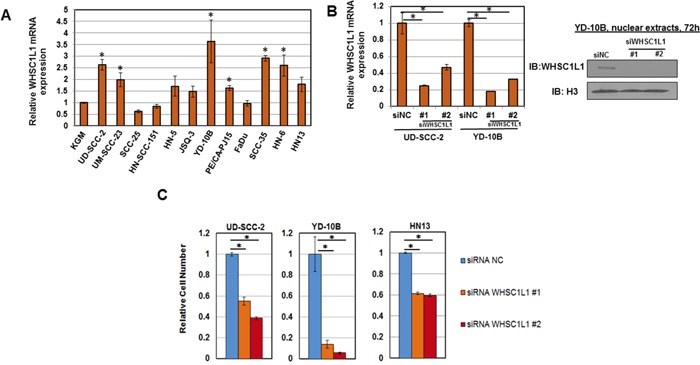
WHSC1L1 is overexpressed in SCCHN cell lines and its knockdown decreases cell viability **A.** WHSC1L1 mRNA expression levels in a panel of 12 SCCHN cell lines. A normal keratinocyte cell line (KGM) was used as a control and mRNA levels were normalized to *GAPDH* and *SDH* mRNA levels. 6 out of 12 SCCHN cell lines showed significant increase in WHSC1L1 mRNA levels compared to KGM cells. * denotes statistically significant difference in mRNA levels in SCCHN cells compared to KGM cells (*P*<0.05, Student's *t*-test, data represented as mean+/−SEM). **B.** RT-PCR confirming knockdown of WHSC1L1 mRNA levels after 72h of treatment with a control siRNA (siNC) and two WHSC1L1-specific siRNAs (siWHSC1L1#1, siWHSC1L1#2) in UD-SCC-2 and YD-10B cells. Western blotting results for WHSC1L1 (162kD isoform) in YD-10B cells are also shown under the same conditions. Nuclear extracts were obtained and histone H3 was used as a loading control. **C.** Proliferation MTT assays in 3 SCCHN cell lines with higher endogenous expression of WHSC1L1. UD-SCC-2 is an HPV-positive cell line and YD-10B and HN13 are HPV-negative cell lines. Cells were plated in 24-well plates in quadruples and were transfected with two WHSC1L1-specific siRNAs, as well as a negative control siRNA (siNC). MTT assays were performed on day 6 of siRNA treatment. Statistically significant reduction of cell viability was observed in the WHSC1L1-specific siRNA-treated cells compared to the siNC-treated cells (* *P*<0.05, Student's *t*-test, data represented as mean+/−SEM).

To assess the expression pattern of WHSC1L1 in SCCHN cell lines, mRNA expression analysis of WHSC1L1 was performed in a panel of 12 SCCHN cell lines (Supplementary Table S1). Significant overexpression of WHSC1L1 was observed in 6 of 12 (50%) SCCHN cell lines compared to a normal keratinocyte cell line (KGM) used as a control (Figure 2A). Western blotting analysis for WHSC1L1 was also performed in 9 SCCHN cell lines and revealed overexpression of WHSC1L1 (162kD) in 2 of 9 SCCHN cell lines (22%) (Supplementary Figure S1). To examine the importance of WHSC1L1 in cell proliferation and survival, RNA interference experiments were conducted in a HPV-positive (UD-SCC-2) and two HPV-negative cell lines (YD-10B and HN13) with endogenous WHSC1L1 overexpression, using two different WHSC1L1-specific siRNAs (siWHSC1L1#1 and siWHSC1L1#2) and a control siRNA (siNC). Knockdown of WHSC1L1 was confirmed at the protein level (Figure 2B, Supplementary Figure S2) and cell viability was measured by an MTT assay (Cell Counting Kit-8) on day 6 of siRNA treatment. Knockdown of WHSC1L1 significantly decreased the viability of both the HPV-positive and the two HPV-negative SCCHN cell lines (Figure 2C). The data above support that WHSC1L1 knockdown decreases the cell proliferation and/or survival of SCCHN cells.

### WHSC1L1 induces H3K36 dimethylation in SCCHN cells

**Figure 3:**
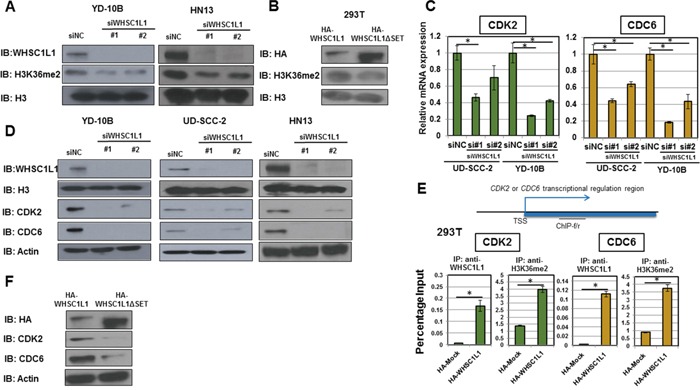
WHSC1L1 induces H3K36me2 dimethylation in SCCHN cells and directly regulates the transcription of cell cycle genes *CDC6* and *CDK2* **A.** Knockdown of WHSC1L1 leads to decrease in global dimethylated H3K36 (H3K36me2) in YD-10B and HN13 cells. YD-10B and HN13 cells were transfected with two WHSC1L1-specific siRNAs (siWHSC1L1#1, siWHSC1L1#2) and with a control siRNA (siNC). After 3 days of transfection, nuclear extracts were obtained and blotted for H3K36me2. H3 was used as a loading control. **B.** Overexpression of HA-WHSC1L1 in 293T cells leads to increase in global H3K36me2 levels compared to HA-WHSC1L1ΔSET (enzyme-inactive WHSC1L1). 293T cells were transfected with HA-WHSC1L1 or HA-WHSC1L1ΔSET vectors, and nuclear extracts were obtained and blotted for H3K36me2. H3 was used as a loading control. **C.** RT-PCR confirming decrease of CDC6 and CDK2 mRNA levels in UD-SCC-2 and YD-10B cells after 3 days of treatment with WHSC1L1-specific siRNAs (*P*<0.05, Student's *t*-test, data represented as mean+/−SEM). **D.** Western blotting confirming decrease of CDC6 and CDK2 protein levels in YD-10B, UD-SCC-2 and HN13 after 3 days of treatment with WHSC1L1-specific siRNAs. **E.** WHSC1L1 transcriptionally regulates the expression of CDK2 and CDC6 through induction of H3K36 dimethylation in the gene body regions of these genes. Chromatin immunoprecipitation (ChIP) assays were performed in a gain-of-function system. 293T cells were transfected with HA-Mock or HA-WHSC1L1 vectors for 48h and ChIP assays using anti-WHSC1L1 and anti-H3K36me2 antibodies were performed with ChIP primers targeting the gene body regions of CDK2 and CDC6 (* *P*<0.05, Student's *t*-test, data represented as mean+/−SEM). **F.** Western blotting for CDK2 and CDC6 in sonicated extracts of 293T cells transfected with wild type versus enzyme-inactive WHSC1L1. The protein levels of CDK2 and CDC6 were decreased in 293T cells overexpressing HA-WHSC1L1-ΔSET (enzyme-inactive WHSC1L1) compared to cells overexpressing wild type WHSC1L1. Actin was used as a loading control.

To assess whether WHSC1L1 induces H3K36 dimethylation in SCCHN cells, we performed knockdown experiments of WHSC1L1 in YD-10B and HN13 SCCHN cells using two WHSC1L1 specific siRNAs (siWHSC1L1#1 and siWHSC1L1#2). After 3 days of siRNA treatment, a decrease in dimethylated H3K36 was observed in both cell lines treated with WHSC1L1 specific siRNAs compared to control siRNA (Figure 3A). To validate this finding in an overexpression system, 293T cells with endogenously low expression of WHSC1L1 were transfected with HA-WHSC1L1-WT (wild type) and HA-WHSC1L1-ΔSET (enzyme-inactive WHSC1L1) for 48h, and nuclear extracts were blotted for dimethylated H3K36. Results showed higher levels of dimethylated H3K36 protein levels in 293T cells transfected with the wild type WHSC1L1 than those with enzyme-inactive WHSC1L1 (Figure 3B), indicating that WHSC1L1 regulates the levels of H3K36 dimethylation in SCCHN cells.

### WHSC1L1 regulates the transcription of cell cycle genes *CDK2* and *CDC6* through H3K36 dimethylation

To identify genes regulated by WHSC1L1 in SCCHN cells, we performed a cDNA microarray in two SCCHN cell lines (UD-SCC-2, YD-10B) after treatment of cells with a control siRNA (siNC) and two WHSC1L1 specific siRNAs (siWHSC1L1#1 and siWHSC1L1#2). cDNA microarray analysis revealed significant downregulation of 93 genes by more than 50% in both UD-SCC-2 and YD-10B cells after knockdown of WHSC1L1 (Supplementary Table S2). Interestingly, among these we found four genes necessary for progression of the cell cycle from the G1 to the S phase, cell division cycle 6 (*CDC6*), cyclin-dependent kinase 2 (*CDK2*), cyclin D1 (*CCND1*) and cell division cycle 25A (*CDC25A*). KEGG (Kyoto Encyclopedia of Genes and Genomes) pathway analysis confirmed enrichment in the “cell cycle” function (Supplementary Figure S3). CDC6 is a protein kinase that is required for the initiation of DNA replication [9] and has been shown to promote the development of malignant behavior and DNA hyperreplication [10, 11]. CDK2 is a serine/threonine protein kinase which binds to cyclin E and promotes the E2F transcriptional program, thus allowing transition to the S phase [12]. CCND1 is a well known oncogene which forms a complex and functions as a regulator of CDK4 and CDK6, mediating G1 to S phase transition. CDC25A is a dual-specificity protein phosphatase of cyclin-dependent kinases CDK2, CDK4 and CDK6 (CDKs) which positively regulates their activity towards G1 to S phase progression [13]. Given the frequent deregulation of these genes in cancer and their necessary role in DNA replication, they have attracted significant attention as potential anticancer targets.

We further validated the expression levels of CDC6, CDK2, CCND1 and CDC25A as potential downstream targets of WHSC1L1 by performing quantitative real-time PCR in UD-SCC-2 and YD-10B cells (Figure 3C, Supplementary Figure S4). We confirmed downregulation of CDC6 and CDK2 at the mRNA, as well as at the protein level in three SCCHN cell lines, YD-10B, UD-SCC-2 and HN13 cells, transfected with two WHSC1L1 specific siRNAs (Figure 3D). However, we could not confirm decrease of CCND1 and CDC25A at the protein level for either cell line. Based on the above, we decided to concentrate our further analysis on CDC6 and CDK2.

To evaluate whether WHSC1L1 directly regulates the transcription of *CDC6* and *CDK2* through H3K36 dimethylation, we performed chromatin immunoprecipitation (ChIP) assays using ChIP-grade antibodies for WHSC1L1 and H3K36me2, targeting the gene body of each of *CDC6* and *CDK2* with 4 different primer sets. To this purpose, we used a gain-of-function system of 293T cells which were transfected with HA-Mock and HA-WHSC1L1 (wild type) vectors for 48h. CDC6 levels in WHSC1L1-immunoprecipitants were increased by 42 times in the HA-WHSC1L1 wild type transfected 293T cells compared with HA-Mock cells (*P*<0.0001, Student's t-test). Similarly, a 4-fold increase of CDC6 was observed in the H3K36me2-enriched cell lysates (*P*=0.0002, Student's *t*-test). We also observed an increase in the CDK2 levels by 26 times in the WHSC1L1-immunoprecipitants of the lysates from HA-WHSC1L1 wild type transfected 293T cells (*P*=0.01, Student's t-test), and by approximately 3 times in the H3K36me2-immunoprecipitants (*P*=0.0005, Student's t-test) (Figure 3E). These results support that both *CDC6* and *CDK2* are transcriptionally regulated directly by WHSC1L1 through induction of H3K36 dimethylation in SCCHN cells. Consistently, the protein levels of CDK2 and CDC6 were decreased in 293T cells overexpressing HA-WHSC1L1-ΔSET (enzyme-inactive WHSC1L1) compared to cells overexpressing wild type WHSC1L1 (Figure 3F), further indicating that the methyltransferase activity of WHSC1L1 is necessary for the transcriptional regulation of *CDC6* and *CDK2* by WHSC1L1.

### WHSC1L1 knockdown induces marked G1/S phase arrest through transcriptional regulation of CDK2 and CDC6

**Figure 4:**
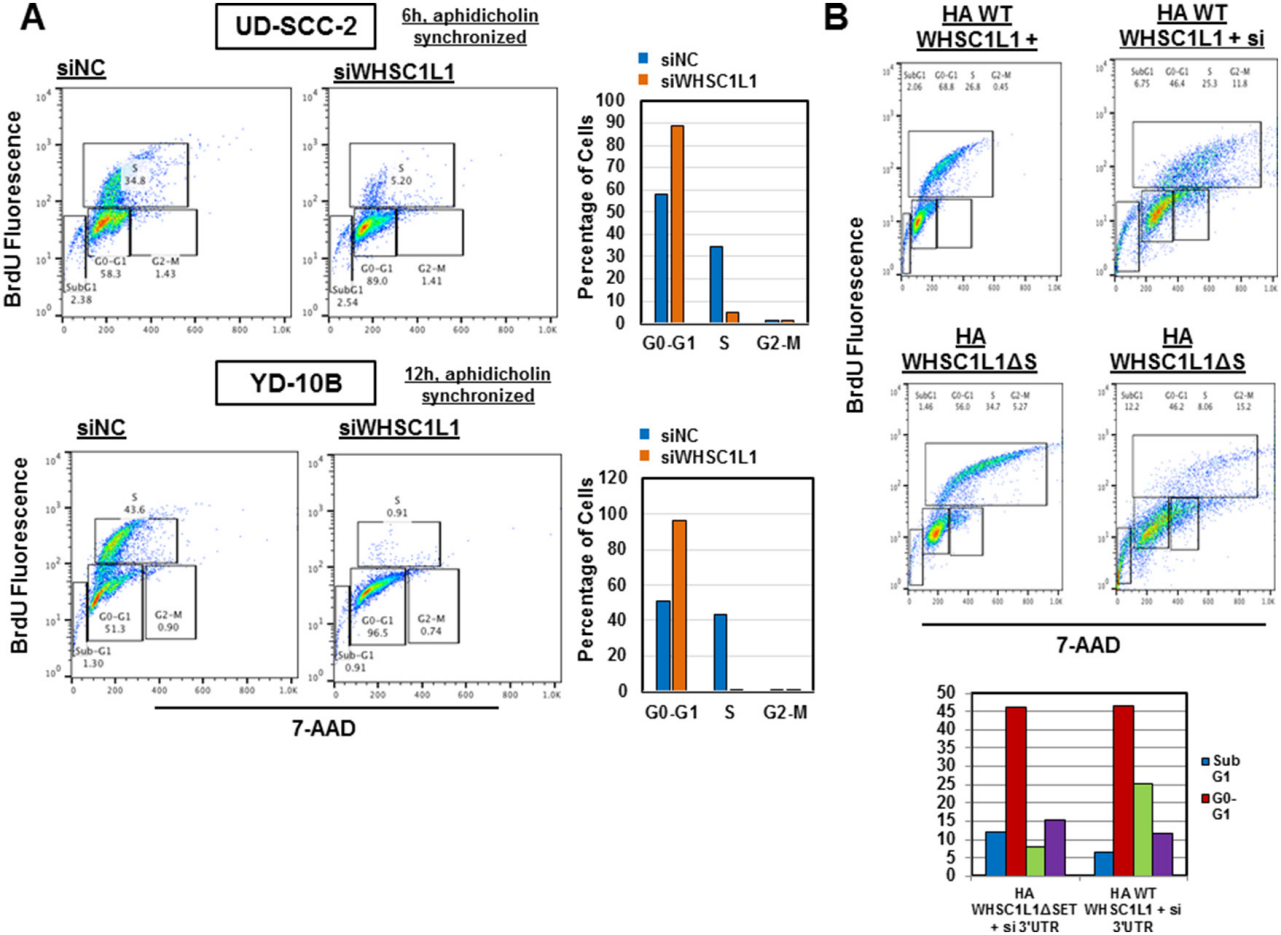
WHSC1L1 knockdown significantly suppresses transition of the cell division cycle from G1 to S phase in SCCHN cells **A.** UD-SCC-2 and YD-10B cells were transfected with siNC and siWHSC1L1#1 and cell cycle was synchronized using aphidicholin (5μg/mL) (48h exposure) after 24h of siRNA transfection. At 72h of transfection, the cell cycle was released and flow cytometry was performed using the BD Pharmigen cell cycle flow cytometry kit. Results are shown for 6h and 12h after cell cycle release for UD-SCC-2 and YD-10B cells respectively. In UD-SCC-2 cells, S phase cells were decreased from 35% to 5%, with a concordant increase in the G0-G1 cells from 58% to 89%. In YD-10B cells, S phase cells were decreased from 44% to 1% and G0-G1 cells were increased from 51% to 96%. These results were reproduced in duplicates with unsynchronized cell cycle analysis. **B.** YD-10B cells were transfected with a siRNA targeting the 3′ untranslated region (3′UTR) of the long WHSC1L1 isoform for 72h. At 24h of siRNA transfection, cells were transfected with HA-WHSC1L1 WT or enzyme-inactive HA-WHSC1L1ΔSET vectors for 48h. Cell cycle flow cytometry was performed after 72h of siRNA transfection. Results revealed that suppression of the S phase occurred in the si# 3′UTR cells transfected with the HA-WHSC1L1ΔSET vector, but was recovered in cells transfected with the HA-WHSC1L1 WT vector. Histographic representation of these results is included.

Given the critical function of CDK2 and CDC6 in the transition from G1 to S phase of the cell division cycle, we sought to determine whether WHSC1L1 knockdown leads to arrest of the cell cycle progression from G1 to S phase. To investigate this hypothesis, we transfected UD-SCC-2 and YD-10B cells with control siRNA (siNC) and a WHSC1L1 specific siRNA (siWHSC1L1#1) for 72h and synchronized the cell cycle at the G0/G1 phase with 48 hours of aphidicholin incubation. After releasing the cell cycle, we performed flow cytometric analysis at 6h and 12h which revealed a marked decrease of the S phase and a concordant increase in the G0/G1 phase cells in both cell lines treated with siWHSC1L1 compared with those treated with control siRNA (Figure 4A). To further validate that this phenotype was due to knockdown of WHSC1L1 and was not mediated by an off-target effect of the siRNA treatment, we then performed a rescue experiment by transfecting YD-10B cells with an siRNA targeting the 3′-untranslated region of WHSC1L1 (3′UTR) which was confirmed to knock down the long WHSC1L1 isoform (Supplementary Figure S5). At 24h of the 3′UTR WHSC1L1 siRNA transfection, we transduced the cells with HA-WHSC1L1 wild type vector, of which the WHSC1L1 transcript did not correspond to the 3′UTR siRNA, versus an enzyme-inactive HA-WHSC1L1ΔSET vector for 48h. Flow cytometric analysis at 72h revealed that the percentage of S-phase cells was decreased from 35% in the siNC group transfected with HA-WHSC1L1ΔSET to 8% in the si3′UTR group transfected with HA-WHSC1L1ΔSET, but only from 27% to 25% in the siNC/HA-WHSC1L1 group compared to the si3′UTR/HA-WHSC1L1 group. These findings support that the HA-WHSC1L1 wild type vector, but not the enzyme-inactive one, rescued the S phase progression in the cells transfected with the si3′UTR WHSC1L1 (Figure 4B). The observation that there was no rescue of the S phase in cells transfected with the enzyme-inactive HA-WHSC1L1ΔSET vector indicates that the methyltransferase activity of WHSC1L1 incurred by the SET domain is necessary for augmentation of the S phase in SCCHN cells. These results support that WHSC1L1 is necessary for the transition of SCCHN cells from the G1 to the S phase through its H3K36 dimethylation activity.

## DISCUSSION

WHSC1L1 is a protein lysine methyltransferase that is recurrently amplified in SCCHN, as well as many other cancer types, such as bladder, breast, and lung squamous cell carcinomas. In addition to SCCHN, WHSC1L1 is frequently amplified in human breast cancer cell lines [14] and in 13% of breast cancer samples [3, 4], and high WHSC1L1 mRNA levels have been associated with worse survival in breast cancer patients [15]. Knockdown of WHSC1L1 in 8p11.23 amplified breast cancer cell lines reduced the growth of cancer cells, and concordantly overexpression of WHSC1L1 in non-transformed mammary epithelial cells led to the formation of disorganized acini [16]. The TCGA database has also described frequent WHSC1L1 amplification in bladder cancer (11%) and squamous cell carcinoma of the lung (21%). Furthermore, the t(8;11) translocation generating the NUP98-NSD3 fusion product has been described in acute myeloid leukemia [17] and in chemotherapy-related myelodysplastic syndrome [18], further suggesting its function as an oncogene. Preclinical studies have underlined the importance of WHSC1L1 in the cell growth and survival of lung, bladder and breast cancer cell lines [7, 19, 20], though the detailed mechanisms of its action are largely unknown. More recently, Shen *et al* [21] showed that BRD4, which is necessary for the maintenance of the malignant phenotype of AML, requires the PWWP domain of the short WHSC1L1 isoform as an adapter protein which links BRD4 to the CHD8 chromatin remodeler, while all of these proteins are co-localized in the AML genome and are released from super-enhancer regions through BET bromodomain chemical inhibition. WHSC1L1 was also reported to interact with BRD4 through the fusion product WHSC1L1-NUT (NSD3-NUT) observed in a rare squamous cell malignancy known as NUT midline carcinoma, while these cells underwent growth arrest and differentiation upon treatment with the bromodomain inhibitor JQ1 [22].

This study has shown that WHSC1L1 is significantly overexpressed in SCCHN compared to normal epithelium, and that 58% of locoregionally advanced SCCHN tumors demonstrated high expression levels. The discrepant percentage of 9.3% amplification frequency of WHSC1L1 in SCCHN implies that additional mechanisms, such as transcriptional upregulation and/or increased protein stability, may contribute to the high expression protein levels of WHSC1L1 in SCCHN tissues. WHSC1L1 expression increased significantly in the transformation process from normal epithelial to dysplastic cells and further to SCCHN cells and is associated with heavier smoking history and poorly differentiated SCCHN. To our knowledge, there is only one study [19] that assessed the association between WHSC1L1 protein levels and various clinicopathological parameters in bladder cancer, but found no correlations, including smoking history and grade. Though tumor grade is not known to affect prognosis, heavy smoking history is associated with worse survival in SCCHN. The association between heavier smoking and increased WHSC1L1 protein expression levels raises an interesting and significant question. A biological possibility to explain this association is that smoking may induce genetic instability which in turn may be associated with gene amplifications, including WHSC1L1 which may be selected due to its oncogenic function. To this purpose, future investigations comparing the 8p11.23 amplification frequency between dysplastic epithelial samples of subjects with heavy versus no smoking history would be worth pursuing.

Furthermore, we have shown that WHSC1L1 knockdown decreases the viability of SCCHN cells. We have also demonstrated that WHSC1L1 induces global H3K36 dimethylation changes in SCCHN cells and it directly regulates the transcription of cell cycle-related genes *CDC6* and *CDK2*, probably through enhancement of H3K36 dimethylation. In accordance with the critical roles of CDC6 and CDK2 in the cell cycle progression to the S-phase, WHSC1L1 knockdown led to marked decrease of the S phase, a phenotype which was rescued with introduction of WHSC1L1, but not with that of enzyme-inactive WHSC1L1. CDC6 is a protein kinase essential for the initiation of DNA replication, in that its ATPase activity is vital for the assembly of the origin recognition complex, CDT1 and MCM2-7 helicase at replication origins to form pre-replication complexes that initiate DNA replication during the G1 phase of the cell division cycle [9, 23]. CDK2 is critical for the transition from the G1 to the S phase by binding to cyclin E and promoting the E2F transcriptional program and the initiation of DNA synthesis [12]. Inhibition of CDK2 in ovarian cancer cells overexpressing cyclin E has also been shown to significantly suppress cancer cell proliferation [20]. The above underline the necessity and importance of these cell cycle genes in the proliferation of cancer cells.

Taking the above into consideration, as well as the very low expression levels of WHSC1L1 in normal tissues [19], WHSC1L1 may serve as a promising candidate for drug development for patients with SCCHN as well as other cancer types characterized by WHSC1L1 amplification or overexpression.

## MATERIALS AND METHODS

### Immunohistochemistry in head and neck cancer tissue microarrays

The expression pattern of WHSC1L1 in 154 SCCHN, 19 dysplastic and 18 normal epithelial tissue sections were examined by immunohistochemistry. SCCHN sections were derived from biopsies of patients with local or locoregionally advanced disease previous to treatment with either surgery with or without adjuvant chemoradiation, or definitive chemoradiation. Slides of paraffin-embedded squamous cell carcinoma tumor specimens, dysplastic and normal epithelial tissues were deparaffinized, rehydrated and sections were treated with antigen retrieval buffer (pH 6, S2367, DAKO, Carpinteria, CA) in a steamer for 20 min at 96°C. Anti-WHSC1L1 antibody (11345-1-AP, Proteintech, Rosemont, IL, dilution 1:400) was applied on tissue sections for one hour incubation at room temperature. Following TBS wash, the antigen-antibody binding was detected with the Bond Refine polymer detection system (DS9800, Leica Biosystems, Wetzlar, Germany) and DAB+ chromogen (DAKO, K3468). Tissue sections were briefly immersed in hematoxylin for counterstaining and were covered with cover glasses. An expert head and neck cancer pathologist and an additional reviewer blinded to clinical outcomes performed semi-quantitative analysis of WHSC1L1 staining using a four-grade scale defined as follows: negative, grade 0; mild, grade +1; moderate, grade +2; and strong staining intensity, grade +3. This methodology was chosen based on the observation that WHSC1L1 staining was observed to be homogeneous in each tissue sample. Use of tissues was approved by the University of Chicago Institutional Review Board (IRB 12-2125 and IRB 12-2117).

### Cell culture

Squamous cell carcinoma cell lines UD-SCC2, SCC-23, SCC-25, SCC-35, HN-SCC-151, PE/CA-PJ15, FaDu, JSQ-3, HN-5, HN-6, HN13, YD-10B were derived from patients with locoregionally advanced SCCHN and were kindly provided by Dr. Tanguy Seiwert (University of Chicago). Detailed characteristics of each cell line are shown in Supplementary Table S1. UD-SCC-2, SCC-23, SCC-25, SCC-35, HN-SCC-151 and JSQ-3 were maintained in DMEM/F12 medium, 10% fetal bovine serum, 1% penicillin/streptomycin and 2 nM L-glutamine. PE/CA-PJ15 was maintained in IDMEM, 10% fetal bovine serum, 1% penicillin/streptomycin and 2 nM L-glutamine. HN-5, HN-6 and HN13 cells were maintained in DMEM medium with 10% fetal bovine serum, 1% penicillin/streptomycin, and 2 nM L-glutamine. FaDu cells were maintained in RPMI medium, 10% fetal bovine serum, 1% penicillin/streptomycin and 2 nM L-glutamine. KGM cells (normal human keratinocytes, 00192627; Lonza, Basel, Switzerland) were maintained in KGM-Gold keratinocyte growth medium supplemented by BPE, transferrin, insulin, hEGF, hydrocortisone, epinephrine, transferrin and gentamicin/amphotericin B (KGM Gold Bullet kit 00192060). Human embryonic kidney 293T cells were maintained in DMEM medium with 10% fetal bovine serum and 1% penicillin/streptomycin.

### Expression vector construction

An entire coding sequence of WHSC1L1 (GenBank NCBI Reference Sequence: NM_023034.1) was amplified from human testis cDNAs using KOD-Plus-Neo (TOYOBO, Osaka, Japan) DNA polymerase and cloned into pCAGGSn3FC vector between *NotI* and *XhoI* restriction enzyme sites (pCAGGS-WT WHSC1L1-HA). To prepare an enzyme-inactive WHSC1L1, the coding sequence of the SET domain was deleted from the entire coding sequence of WHSC1L1 (pCAGGS-WHSC1L1-ΔSET-HA).

### Quantitative real-time PCR

Specific primers for human *GAPDH* (housekeeping gene), *SDH* (housekeeping gene), *WHSC1L1*, *CDK2*, *CDC6* were designed (primer sequences in Supplementary Table S3). PCR reactions were performed using ViiA 7 real-time PCR system (Thermo Fisher Scientific, Waltham, MA) following the manufacture's protocol.

### Western blotting

Nuclear extracts were prepared using the Nuclear Extract kit (Active Motif) to examine protein levels of WHSC1L1, and cytoplasmic extracts using the NE-PER nuclear and cytoplasmic extraction kit (78833, ThermoFisher Scientific) were obtained to examine protein levels of cytoplasmic CDK2, CDC6 and ACTB. Primary antibodies used were anti-WHSC1L1 (11345-1-AP, Proteintech, dilution: 1:5000), anti-CDK2 (#2546, Cell Signaling Technology, Danvers, MA, dilution: 1:1000), anti-CDC6 (#3387, Cell Signaling Technology, Danvers, MA, 1:1000), anti-ACTB (A5441, Sigma-Aldrich, St. Louis, MO, dilution: 1:5000) and anti-HA (H6908, Sigma-Aldrich, St. Louis, MO, dilution 1:2000). For detection of histone marks, nuclear extracts were prepared using the Nuclear Extract kit (Active Motif) and 2.5 μg of each extract was loaded for each experiment. Antibodies used were anti-H3K36me2 (07-369, Merck Millipore, dilution: 1:10000), and anti-H3 (ab1791, Abcam, Cambridge, UK, dilution: 1:50000).

### siRNA transfection and cell growth assays

MISSION_ siRNA oligonucleotide duplexes were purchased from Sigma–Aldrich for targeting the human WHSC1L1 transcripts (SASI_Hs01_00082045 and SASI_Hs01_00082044). siNegative control (siNC), which consists of three different oligonucleotide duplexes, were used as control siRNAs (Cosmo Bio, Tokyo, Japan). The siRNA sequences are described in Supplementary Table S3. SCCHN cells were plated overnight in 24-well plates (2-4 × 10^4^ cells/well) and were transfected with siRNA duplexes (50 nM final concentration) using Lipofectamine RNAimax (Thermo Fisher Scientific) for 144 h (6 days) with retransfection performed at day 4. The number of viable cells was measured using the Cell Counting Kit-8 (Dojindo, Kumamoto, Japan) on day 6 [24].

### ChIP assays

ChIP assays were performed using ChIP Assay kit (17-295, Merck Millipore) according to the manufacture's protocol. Briefly, WHSC1L1 and fragmented chromatin complexes were immunoprecipitated with anti-WHSC1L1 (11345-1-AP, Proteintech, dilution: 1: 50) and anti-H3K36me2 (07-369, Merck Millipore, dilution: 1:100) antibodies 48 h after transfection of YD-10B cells with siRNAs for WHSC1L1 (SASI_Hs01_00082045). The same procedure was followed in 293T cells transfected with HA-Mock and HA-WHSC1L1 vectors for 48h. DNA fragments were quantified by real-time PCR using CDK2-ChIP forward primer (5′-TGG TGG CGG TCG GGA ACT CGG TG-3′) and reverse (5′-GGA AAC AGG GCC CAG TAC CGT GG-3′) primers, CDC6-ChIP forward (5′-GAG CGC GGC TGG AGT TTG CTG CTG-3′) and reverse (5′-CAG GAT CCT TCT CAC GTC TCT CAC-3′) primers, and CCND1-ChIP forward (5′-CAC ACG GAC TAC AGG GGA GTT TTG-3′) and reverse (5′-GGA GCG TGC GGA CTC TGC TGC TCG-3′) primers.

### Cell cycle analysis assays

The 5-bromo-20-deoxyuridine (BrdU) flow kit (BD Biosciences, San Jose, CA) was used to determine the cell cycle kinetics. The assay was performed according to the manufacturer's instructions. Briefly, cells were seeded overnight in 10cm tissue culture dishes and treated with siRNAs targeting WHSC1L1 (50 nM, SASI_Hs01_00082045) described as above using medium with 10% FBS without antibiotics for 72h, followed by addition of 10 μM BrdU. At 24h of transfection, medium was suctioned and repleted with medium containing aphidicholin (5μg/mL) for 48h to achieve cell cycle synchronization at the G0/G1 phase. At 72h, cells were released from aphidicholin and were exposed to BrdU at different cell cycle time points (0h, 6h, 12h, 24h). Cells were then fixed in a solution containing paraformaldehyde and saponin. Then samples were incubated with DNAase for 1 h at 37°C and FITC-conjugated anti-BrdU antibody (dilution: 1:50) was added for 20 min at room temperature. Total DNA was stained with 7-amino-actinomycin D (7-AAD), followed by flow cytometric analysis. This analysis was performed in YD-10B and UD-SCC-2 cells.

### Microarray hybridization and statistical analysis

UD-SCC2 and YD-10B cells were plated in 10cm dishes and treated with two different WHSC1L1 specific siRNAs (50nM, SASI_Hs01_00082045 and SASI_Hs01_00082044) and a negative control siRNA (siNC) for 48h. Purified total RNA isolated from these samples was labeled and hybridized onto Affymetrix GeneChip U133 Plus 2.0 oligonucleotide arrays (Affymetrix, Santa Clara, CA) according to the manufacturer's instructions (33-35). Probe signal intensities were normalized by Affymetrix *MAS5* algorithm (Expression Console™ Software). In order to compare the signal intensity between two different samples with accuracy, we only used the data whose *P*-value is less than 0.05; up-regulation means the intensity ratio is more than 3 and down-regulation means the intensity ratio is less than 0.5.

### Statistical analysis

Spearman rank correlation coefficients were calculated to assess associations between WHSC1L1 and WHSC1 expression levels (using the 4-point IHC scale). Comparison of WHSC1L1 expression levels were compared among tumor, dysplasia, and normal samples using the Wilcoxon rank sum test and the Cochran-Armitage trend test. Clinicopathological correlations were performed in a retrospective manner. Expression levels were dichotomized as 0-1 vs. 2-3 and logistic regression models fit to examine the association between IHC score and gender, age, smoking history, stage, T-stage, N-stage, grade, and HPV status. Cox regression [25] was performed to examine whether WHSC1L1 was prognostic for overall or progression-free survival. Student's *t*-test was performed to compare continuous outcome variables in different groups from the cell line experiments.

## SUPPLEMENTARY FIGURES AND TABLES




