# Supplementary material for: WHSC1L1 drives cell cycle progression through transcriptional regulation of CDC6 and CDK2 in squamous cell carcinoma of the head and neck
Source: Oncotarget. 2016 Jun 7;7(27):42527–38. doi: 10.18632/oncotarget.9897 (PMC5173153; doi:10.18632/oncotarget.9897)
Supplement: Supplementary file 1 [file oncotarget-07-42527-s001.pdf]

WHSC1L1 drives cell cycle progression through transcriptional regulation of CDC6 and CDK2 in squamous cell carcinoma of the head and neck

SUPPLEMENTARY FIGURES AND TABLES

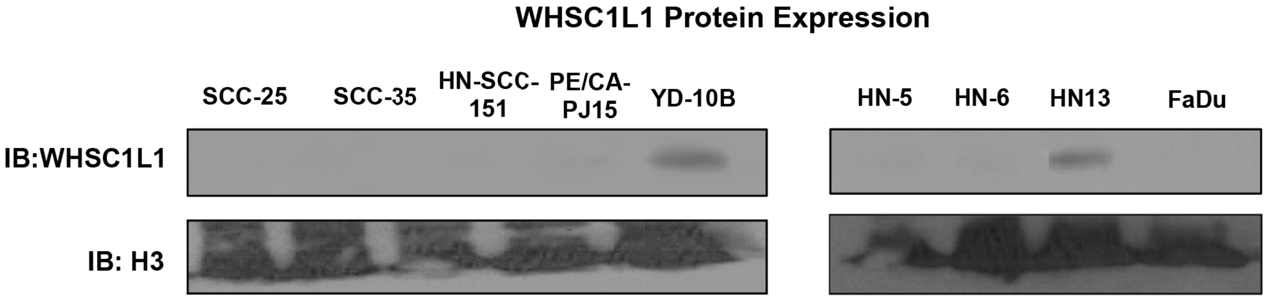

Supplementary Figure S1: WHSC1L1 protein expression levels in 9 SCCHN cell lines. Nuclear extracts were obtained from 9 SCCHN cell lines using the Active Motif kit. 10ug of nuclear extracts were loaded. H3 was used as a loading control.

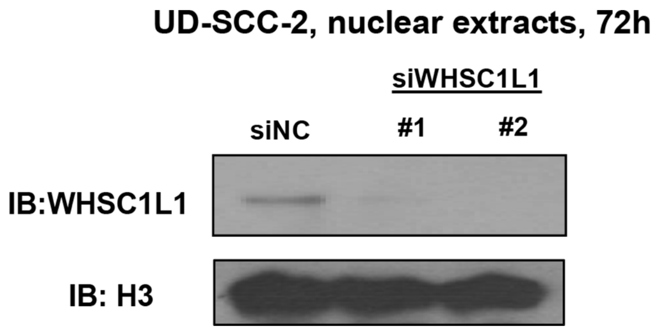

Supplementary Figure S2: WHSC1L1 protein expression levels in UD-SCC-2 cells after WHSC1L1-specific siRNA treatment for 72h. Nuclear extracts were obtained using the Active Motif kit. 10ug of nuclear extracts were loaded. H3 was used as a loading control.

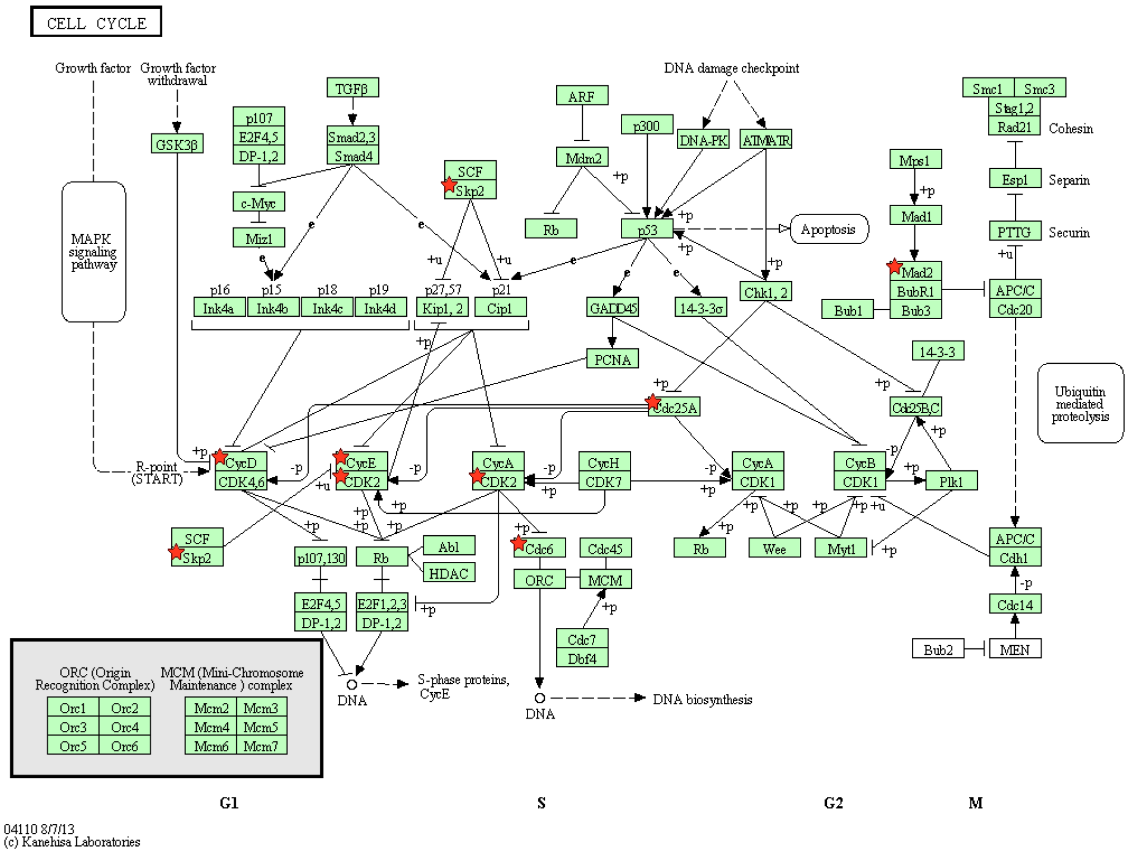

Supplementary Figure S3: KEGG pathway analysis of cDNA microarray results in YD-10B and UD-SCC-2 cells after 48h of WHSC1L1 knockdown with WHSC1L1 specific siRNAs. Enrichment was observed in the "cell cycle" function.

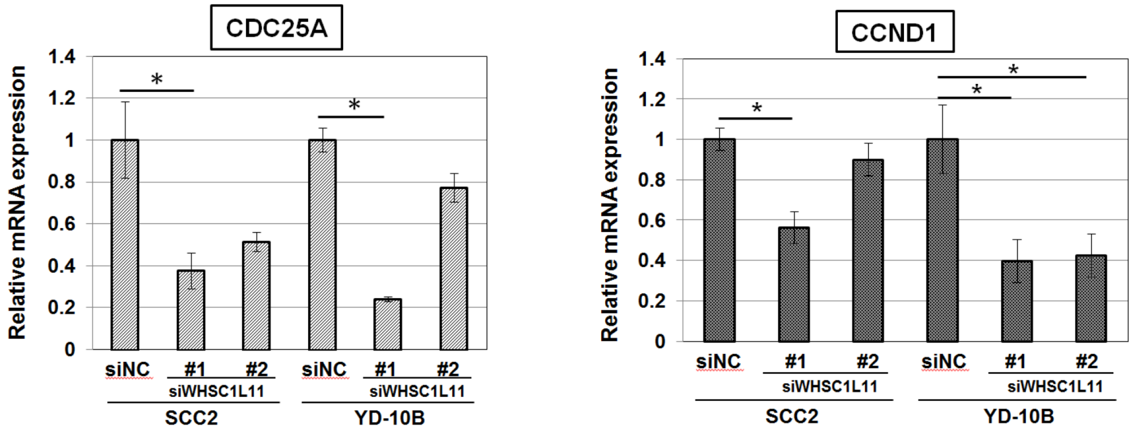

Supplementary Figure S4: RT-PCR confirming decrease of CDC25A and CCND1 mRNA levels in UD-SCC-2 and YD-10B cells after 3 days of treatment with WHSC1L1- specific siRNAs (*P*<0.05, Student's *t*-test, data represented as mean+/-SEM).

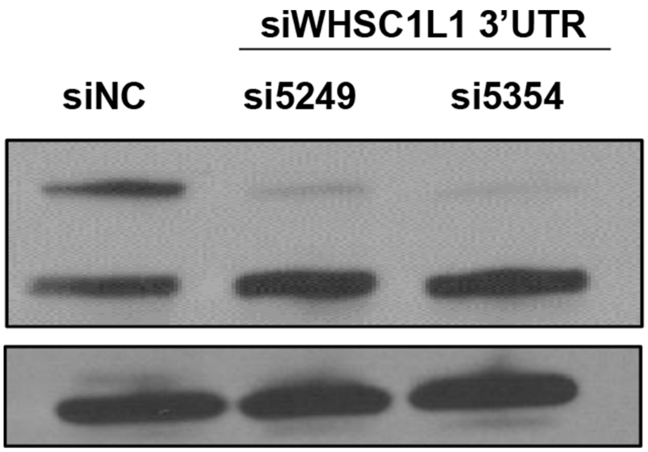

Supplementary Figure S5: WHSC1L1 knockdown with WHSC1L1 siRNAs targeting the 3' untranslated region of the long WHSC1L1 isoform. Note that the short WHSC1L1 is not knocked down with these 3'UTR siRNAs.

Supplementary Table S1: Characteristics of SCCHN cell lines

Cell name	TNM stage	Specimen site	Gender	HPV status
UD-SCC-2	T1N2M0	Hypopharynx	Male	HPV16-positive
UM-SCC-23	T3N0M0	Larynx	Female	HPV16-negative
SCC-25	T2N1M0	Oral cavity	Male	HPV16-negative
SCC-35	T4N0M0	Hypopharynx	Male	HPV16-negative
HN-SCC-151	T3N0M0	Oral tongue	-	HPV16-negative
PE/CA-PJ15	-	Oral tongue	Male	HPV16-negative
FaDu	-	Hypopharynx	Male	HPV16-negative
JSQ-3	T3N0M0	Nasal vestibule	Male	HPV16-negative
HN-5	T2N0M0	Oral cavity	Male	HPV16-negative
HN-6	T2N0M0	Oral cavity	Male	HPV16-negative
HN13	-	Head and Neck	-	-
YD-10B	-	Oral tongue	Male	HPV-negative

Supplementary Table S2: Cumulative table of 93 genes downregulated by WHSC1L1 knockdown (cDNA microarray results)

See Supplementary File 1

Supplementary Table S3: Primer sequences for WHSC1L1, GAPDH, SDH, WHSC1L1, CDC6 and CDK2, and siRNA sequences for siNC, siWHSC1L1#1 and 2

Gene Name	Primer Sequence
GAPDH-f	5’ GCAAATTCCATGGCACCGTC 3’
GAPDH-r	5’ TCGCCCCACTTGATTTTGG 3’
SDH-f	5’ TGGGAACAAGAGGGCATCTG 3’
SDH-r	5’ CCACCACTGCATCAAATTCATG 3’
CDC6-f	5’ TGCTGCAGTTCAATTCTGTGC 3’
CDC6-r	5’ ATAGCTCTCCTGCAAACATCCAG 3’
CDK2-f	5’ CATCTTTGCTGAGATGGTGACTC 3’
CDK2-r	5’ GTAACTCCTGGCCACACCAC 3’
WHSC1L1-f	5’ AGAACGTGCTCAGTGGGATATTGG 3’
WHSC1L1-r	5’ TGCTTGGGATAAAGCCTCTTCAGG 3’

siRNA Name	Sequence
siNegative Control (siNC)	Target#1 Sense: 5’ AUCCGCGCGAUAGUACGUA 3’<br>Antisense: 5’ UACGUACUAUCGCGCGGAU 3’<br>Target#2 Sense: 5’ UUACGCGUAGCGUAAUACG 3’<br>Antisense: 5’ CGUAUUACGCUACGCGUAA 3’<br>Target#3 Sense: 5’ UAUUCGCGCGUAUAGCGGU 3’<br>Antisense: 5’ ACCGCUAUACGCGCGAAUA 3’
siWHSC1L1#1	Sense:5’ GAGAGUAUAAAGGUCAUAA 3’<br>Antisense: 5’ UUAUGACCUUUAUACUCUC 3’
siWHSC1L1#2	Sense: 5’ CUCAUUGACUCCGCCAACA 3’<br>Antisense: 5’ UGUUGGCGGAGUCAAUGAG 3’
siWHSC1L1 3’UTR (si5354)	Sense: 5' GCAGUAGGUAGGCUCACUU 3’<br>Antisense: 5' AAGUGAGCCUACCUACUGC 3’
