# Supplementary material for: WHSC1L1 drives cell cycle progression through transcriptional regulation of CDC6 and CDK2 in squamous cell carcinoma of the head and neck
Source: Oncotarget. 2016 Jun 7;7(27):42527–38. doi: 10.18632/oncotarget.9897 (PMC5173153; doi:10.18632/oncotarget.9897)
Supplement: Supplementary file 2 [file oncotarget-07-42527-s002.docx]

**Table S2.** Cumulative table of 93 genes downregulated by WHSC1L1 (cDNA microarray results).


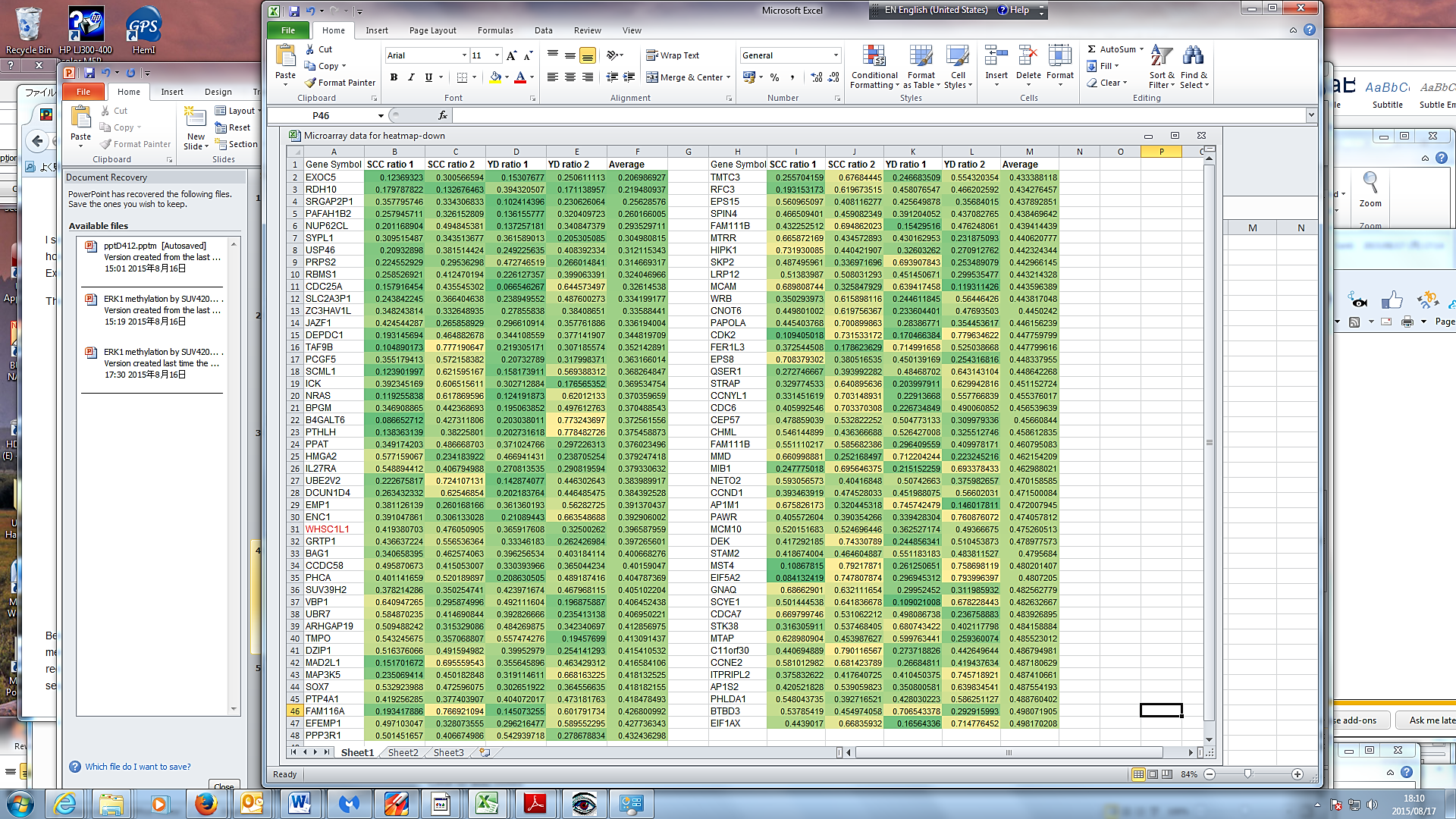


**siWHSC1L1**

**#1**

**siWHSC1L1**

**#2**

**siWHSC1L1**

**#1**

**siWHSC1L1**

**#2**

**Average**

**UD-SCC-2**

**YD-10B**


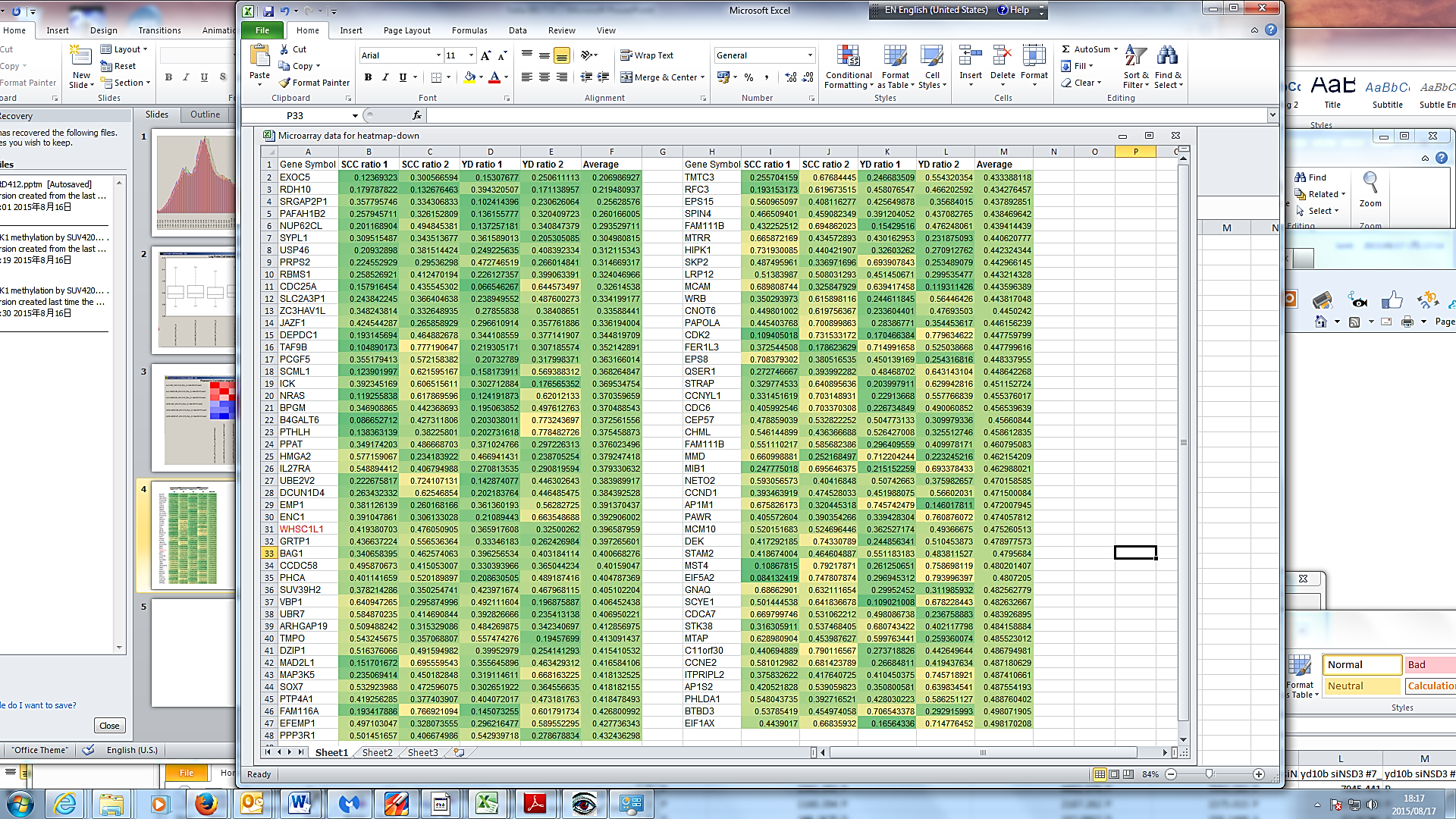


**siWHSC1L1**

**#1**

**siWHSC1L1**

**#2**

**siWHSC1L1**

**#1**

**siWHSC1L1**

**#2**

**Average**

**UD-SCC-2**

**YD-10B**
